# The Efficacy of the Collaborative Respiratory Assessment Score (CoRAS) in Predicting Pneumonia Among Stroke Patients in Kaifukuki Rehabilitation Wards

**DOI:** 10.7759/cureus.79604

**Published:** 2025-02-25

**Authors:** Takeshi Inoue, Takashi Kodama, Tomohiko Takenaka, Shinta Uchida, Kyohei Miura, Shinya Onizuka

**Affiliations:** 1 General Practice, Nagasaki Rehabilitation Hospital, Nagasaki, JPN; 2 General Practice, Kurashiki Medical Center, Kurashiki, JPN; 3 Rehabilitation, Nagasaki Rehabilitation Hospital, Nagasaki, JPN

**Keywords:** kaifukuki rehabilitation ward, multidisciplinary collaboration, pneumonia, post stroke recovery, prediction score

## Abstract

Objective

This study aims to assess the predictive accuracy and clinical utility of the Collaborative Respiratory Assessment Score (CoRAS) in identifying pneumonia risk among stroke patients in Kaifukuki Rehabilitation Wards (KRWs). CoRAS, developed by a multidisciplinary team, incorporates eight clinical parameters to quantify respiratory risk. The study retrospectively applies CoRAS to a cohort of stroke patients and evaluates its predictive performance using statistical methods, including logistic regression and receiver operating characteristic (ROC) curve analysis. Additionally, the study compares CoRAS with traditional risk factors such as age and pre-stroke care level to determine its unique contribution to pneumonia prediction. The findings aim to validate CoRAS as an effective tool for early risk stratification, supporting multidisciplinary collaboration and targeted interventions in KRWs.

Methods

We devised CoRAS as a scoring system based on eight clinical parameters: consciousness level, SpO₂, need for suctioning, history of respiratory diseases, FILS (Food Intake Level Scale), Hoffer criteria, nutritional status, and OHAT-J (Oral Health Assessment Tool - Japanese version). These parameters were weighted proportionally to sum up to a total score of 100. Assessments were independently conducted by seven multidisciplinary professionals, and the score was retrospectively applied to data from 629 stroke patients admitted to our hospital. Data on pneumonia occurrence were collected and analyzed.

Results

Pneumonia was observed in 48 (7.6%) of the 629 patients. The highest variance inflation factor (VIF) among the eight parameters was 2.11, validating the application of a linear combination model. ROC analysis showed an area under the curve of 0.860. Logistic regression revealed that CoRAS had an adjusted odds ratio of 1.06 per point for predicting pneumonia.

Conclusion

CoRAS demonstrated satisfactory predictive validity for pneumonia onset among stroke patients in the KRW, suggesting its utility as an assessment tool for multidisciplinary teams.

## Introduction

The Kaifukuki Rehabilitation Ward (KRW), also referred to as a Convalescent Rehabilitation Ward, is a distinct healthcare system in Japan, focusing on intensive rehabilitation for patients recovering from acute treatment. In KRWs, multidisciplinary teams, including physicians, therapists, nurses, registered dietitians, and dental hygienists, work collaboratively to develop individualized recovery plans tailored to patients’ needs.

Pneumonia is a common complication among patients during their KRW admission. When pneumonia occurs, it can lead to physical deterioration, delayed rehabilitation progress, and even transfer to acute care hospitals, adversely affecting patient outcomes. To prevent pneumonia, it is essential to evaluate patients' risk at an early stage and implement tailored preventive interventions for high-risk individuals [1-12].

Although various scoring systems have been developed to predict pneumonia risk, most target acute care settings and do not consider the unique characteristics of KRW patients. KRWs differ from acute care settings in two key aspects: (1) patients admitted to KRWs often have new disabilities, making their condition distinct from those in the general population, and (2) the team-based care model in KRWs emphasizes multidisciplinary collaboration to support recovery and reintegration into daily life, in contrast to the physician-centered model typical of acute care.

For KRWs to function effectively, it is essential for each professional to share patient conditions and goals, divide tasks, and work in coordination to complement each other. Measures that promote mutual understanding and collaboration among staff members are indispensable. Although some reports have focused on identifying risk factors for pneumonia in KRWs, no scoring system has yet been developed that specifically addresses these aspects [13,14].

At Nagasaki Rehabilitation Hospital (NRH), a multidisciplinary working group developed the Collaborative Respiratory Assessment Score (CoRAS) to assess respiratory risk collaboratively. This study evaluates the predictive accuracy of CoRAS for pneumonia occurrence in stroke patients admitted to KRWs.

## Materials and methods

Study setting

The NRH is a 143-bed facility dedicated exclusively to KRWs. It is located in Nagasaki City, which has a population of approximately 390,000, and provides post-acute care support to acute medical institutions in the region.

Collaborative Respiratory Assessment Score (CoRAS)

A working group comprising 18 professionals from seven disciplines (one physician, six nurses, six physical therapists, one occupational therapist, two speech-language pathologists, one registered dietitian, and one dental hygienist) was established.

The group identified clinical parameters thought to reflect respiratory instability by brainstorming and subsequently using a matrix method to extract items deemed both clinically important and practical to evaluate. Eight parameters were selected: consciousness level, SpO_2_ (as a representative vital sign), need for suctioning and its frequency, physical capability and degree of impairment, swallowing dysfunction, history and number of respiratory diseases, nutritional status, and oral hygiene. Each of the seven professions was responsible for at least one primary parameter.

The evaluation indices for each parameter were selected based on tools already utilized in routine clinical practice across different disciplines. For example, the Japan Coma Scale and suctioning frequency recorded by nurses were used for consciousness level and suctioning needs, respectively. Physical capability was assessed using the Hoffer sitting balance classification [15] by physical and occupational therapists, while swallowing dysfunction was evaluated using the Food Intake Level Scale (FILS) [16] by speech-language pathologists. Respiratory disease history was documented by physicians based on the number of diagnoses. Nutritional status was assessed by registered dietitians using BMI, weight changes, MUST (Malnutrition Universal Screening Tool), and GLIM (Global Leadership Initiative on Malnutrition) criteria [17,18]. Oral hygiene was evaluated using the oral dryness item of the Oral Health Assessment Tool (OHAT-J) [19] by dental hygienists, expressed as "saliva." Each discipline team was responsible for conducting training sessions to ensure standardized assessments and maintain consistency in evaluations. This approach ensured internally valid assessments. Additionally, data completeness was confirmed through systematic data entry checks and verification procedures to ensure no missing data.

All evaluations were completed by the respective professionals within two days of patient admission. Relative weights were assigned to the eight parameters through consensus among group members, resulting in a total score of 100 points (Table 1). Risk categories were also established collaboratively: scores of 0-14 were classified as low risk, 15-39 as moderate risk, and 40-100 as high risk. These thresholds were based on the clinical intuition of the team. CoRAS was implemented on April 27, 2020, with data accessible via the hospital's intranet.

**Table 1:** Scoring criteria for CoRAS parameters The criteria and scoring were determined through discussions within a multidisciplinary working group. CoRAS, Collaborative Respiratory Assessment Score; FILS, Food Intake Level Scale; GLIM, Global Leadership Initiative on Malnutrition; JCS, Japan Coma Scale; MUST, Malnutrition Universal Screening Tool; OHAT-J, Oral Health Assessment Tool - Japanese version

Evaluation Item	Criteria	Score
Consciousness level	Clear	0
	JCS I	3
	JCS II	10
	JCS III	15
SpO₂	≥95%	0
	92–94%	5
	≤91% or requiring oxygen therapy	10
Need for suctioning	None	0
	1–7 times/day	10
	≥8 times/day	20
Sitting balance (Hoffer criteria)	Can sit without hand support	0
	Can sit with hand support	5
	Unable to sit	15
Swallowing dysfunction (FILS)	Level 8–10	0
	Level 7–9	5
	Level 4–6	10
	Level 1–3	15
History of respiratory diseases	None	0
	1	5
	2	8
	≥3	10
Malnutrition (MUST, GLIM)	Normal/overnutrition	0
	At risk of malnutrition	5
	Malnourished	10
Saliva (OHAT-J)	Moist/serous (healthy)	0
	Dry/sticky mucosa (changes present)	3
	Red, parched mucosa (pathological)	5

Study population

Between April 27, 2020, and April 26, 2022, 1,129 patients were admitted to NRH. Of these, 629 patients with a primary admission diagnosis of "ischemic stroke," "intracerebral hemorrhage," or "subarachnoid hemorrhage" (collectively referred to as "stroke") were included in this study. For these 629 patients, CoRAS scores at admission, as well as data on age, sex, pre-stroke care level, and pneumonia occurrence during hospitalization, were retrospectively extracted from medical records.

Pre-stroke care level was categorized based on Japan's public long-term care insurance system, which provides certified care services to elderly and disabled individuals. Patients without prior certification were classified as "no care certification," while those with certification (from support level 1 to care level 5) were classified as "with care certification."

Definitions

Pneumonia was defined as any case requiring intravenous antibiotic treatment during hospitalization and diagnosed in the medical record as "pneumonia," "respiratory infection," "bronchopneumonia," or "aspiration pneumonia."

Analysis procedures

Analysis 1: Baseline Analysis

Age, sex, and pre-stroke care level of patients with pneumonia ("pneumonia group") and without pneumonia ("non-pneumonia group") were compared. Comparisons by risk category were also conducted. Univariate analysis was performed to evaluate the relationship between pneumonia occurrence and the eight CoRAS parameters.

Analysis 2: Relationship Between CoRAS Parameters

Since the eight CoRAS parameters were selected based on the group's discussion, there was a possibility of overlap or redundancy among similar items. This required validation.

Correlation coefficients among the eight parameters were calculated. To check for multicollinearity, variance inflation factors (VIFs) were calculated with pneumonia occurrence as the dependent variable and the eight CoRAS parameters as independent variables.

Analysis 3: Adjusted Logistic Regression Analysis

Given that the absence of multicollinearity was confirmed in analysis 2, CoRAS was treated as a single composite score. Adjusted logistic regression analysis was performed using four factors: CoRAS, age, pre-stroke care level, and sex. Although sex was not significant in the baseline analysis, it was included due to its relevance as a risk factor in previous acute care studies.

Analysis 4: Predictive Accuracy of CoRAS Using ROC Curve Analysis

The contribution of CoRAS to pneumonia prediction was evaluated using receiver operating characteristic (ROC) curves. The study compared three predictive models: model A, which included age, sex, and pre-stroke care level; model B, which utilized the CoRAS score alone; and model C, which incorporated age, sex, pre-stroke care level, and the CoRAS score. Area under the curve (AUC) and Akaike's Information Criterion (AIC) values were calculated for each model, and significant differences between the models were assessed using χ2 tests.

Statistical analysis

Chi-squared tests were used for categorical variables, and Mann-Whitney U tests were used for continuous variables. Statistical significance was set at p < 0.05, and VIFs less than 5 were considered acceptable. Analyses were conducted using R (version 4.3.1) and EZR (version 1.54).

Ethical considerations

This study was conducted with opt-out informed consent and approved by the Nagasaki Rehabilitation Hospital Research Ethics Committee (R4-04).

## Results

Baseline analysis

During the study period, there were no dropouts or missing data. The baseline characteristics of the subjects are summarized in Table 2. Among the 629 stroke patients, 48 (7.6%) developed pneumonia during their hospital stay. There was no significant difference in pneumonia occurrence between males and females; however, a significant difference was observed with age. The pneumonia incidence rates increased progressively with risk category: 0.7% for the low-risk group, 5.9% for the moderate-risk group, and 43.4% for the high-risk group. Significant differences were observed between risk categories. When the low-risk group was used to predict the absence of pneumonia, the sensitivity was 0.49 and the specificity was 0.96. Significant differences in pneumonia occurrence were also observed for pre-stroke care levels. Age and pre-stroke care level were identified as confounding factors for CoRAS, suggesting the need for adjustment when evaluating the predictive accuracy of CoRAS for pneumonia.

**Table 2:** Comparison of characteristics between patients with and without pneumonia A significant difference was observed in age. Pneumonia incidence significantly increased with higher risk categories. When low-risk patients were considered pneumonia-free, sensitivity was 0.49 and specificity was 0.96.

Category	Without Pneumonia	With Pneumonia	p-Value
Total number	581	48	
Sex			
Female	272	20	0.548
Male	309	28	
Age			
Median [IQR]	74 [64.0-82.0]	77 [69.8-84.3]	0.046
Pre-stroke care level			
No certification	433	24	0.001
With certification	148	24	
Risk level			
Low risk	283	2	<0.001
Moderate risk	222	13	
High risk	76	33	

The results of the univariate analysis are presented in Table 3. Among the eight CoRAS parameters, significant differences in pneumonia occurrence were observed for seven parameters, with the exception of SpO₂.

**Table 3:** Comparison of pneumonia incidence across parameters All parameters except SpO₂ were significantly associated with pneumonia incidence.

Parameter	Score	Without Pneumonia	With Pneumonia	p-Value
Consciousness level	0	290	5	<0.001
	3	247	29	
	10	42	10	
	15	2	4	
SpO₂	0	539	41	0.117
	5	30	5	
	10	12	2	
Need for suctioning	0	529	24	<0.001
	10	44	17	
	20	8	7	
Sitting balance	0	277	5	<0.001
	5	192	16	
	15	112	27	
Swallowing dysfunction	0	228	3	<0.001
	5	269	8	
	10	15	6	
	15	69	31	
History of respiratory disease	0	481	25	<0.001
	5	85	19	
	8	14	3	
	10	1	1	
Nutrition	0	329	4	<0.001
	5	125	19	
	10	127	25	
Saliva	0	12	5	<0.001
	3	408	22	
	5	44	21	

Relationship between CoRAS parameters

The analysis results are shown in Table 4. Moderate correlations (r = 0.45-0.65) were observed among consciousness level, suctioning requirement, sitting balance, and swallowing function. The analysis results are shown in Table 5. The maximum VIF among the eight parameters was 2.11. Based on this result, there was no evidence of multicollinearity among the parameters, supporting the validity of using CoRAS as a single composite score.

**Table 4:** Correlation coefficients among CoRAS 8 parameters Moderate correlations (0.45–0.65) were observed among consciousness level, suctioning requirement, sitting balance, and swallowing dysfunction. CoRAS, Collaborative Respiratory Assessment Score

CoRAS Parameters	Consciousness Level	SpO₂	Need for Suctioning	Sitting Balance	Swallowing Dysfunction	History of Respiratory Disease	Malnutrition	Saliva
Consciousness level								
SpO₂	0.23							
Need for suctioning	0.48	0.23						
Sitting balance	0.58	0.23	0.45					
Swallowing dysfunction	0.57	0.18	0.64	0.65				
History of Respiratory disease	0.11	0.27	0.30	0.18	0.26			
Malnutrition	0.40	0.04	0.31	0.40	0.48	0.07		
Saliva	0.35	0.09	0.26	0.39	0.45	0.08	0.33	

**Table 5:** VIF values for each parameter The maximum VIF among the eight parameters was 2.11. VIF, variance inflation factor

Parameter	VIF
Consciousness level	1.60
SpO₂	1.16
Need for suctioning	1.71
Sitting balance	1.83
Swallowing dysfunction	2.11
History of respiratory disease	1.17
Nutrition	1.21
Saliva	1.27

Adjusted logistic regression analysis

The maximum VIF among the four factors (CoRAS, age, pre-stroke care level, and sex) was 1.35, indicating that logistic regression analysis was feasible. The analysis results are presented in Table 6. Pre-stroke care level and CoRAS were significantly associated with pneumonia occurrence. The adjusted odds ratio for age was 1.00 per year, and no significant difference was observed for sex. For pre-stroke care level, the adjusted odds ratio for those with a care certification was 2.19. The adjusted odds ratio for CoRAS was 1.06 per point.

**Table 6:** Odds ratios and VIF for variables The maximum VIF for age, sex, pre-stroke care level, and CoRAS was 1.35. Significant differences were observed for "pre-stroke care level" and "CoRAS." The adjusted odds ratio was 2.19 for patients with care certification and 1.06 per CoRAS point. CoRAS, Collaborative Respiratory Assessment Score; VIF, variance inflation factor

Variable	VIF	Odds Ratio	95% Confidence Interval	p-Value
Age	1.35	1.00	0.97–1.03	0.84
Sex	1.09	1.73	0.87–3.52	0.12
Pre-stroke care level	1.31	2.19	1.02–4.71	0.04
CoRAS	1.02	1.06	1.05–1.08	<0.01

Predictive accuracy of CoRAS using ROC curve analysis

**Figure 1:**
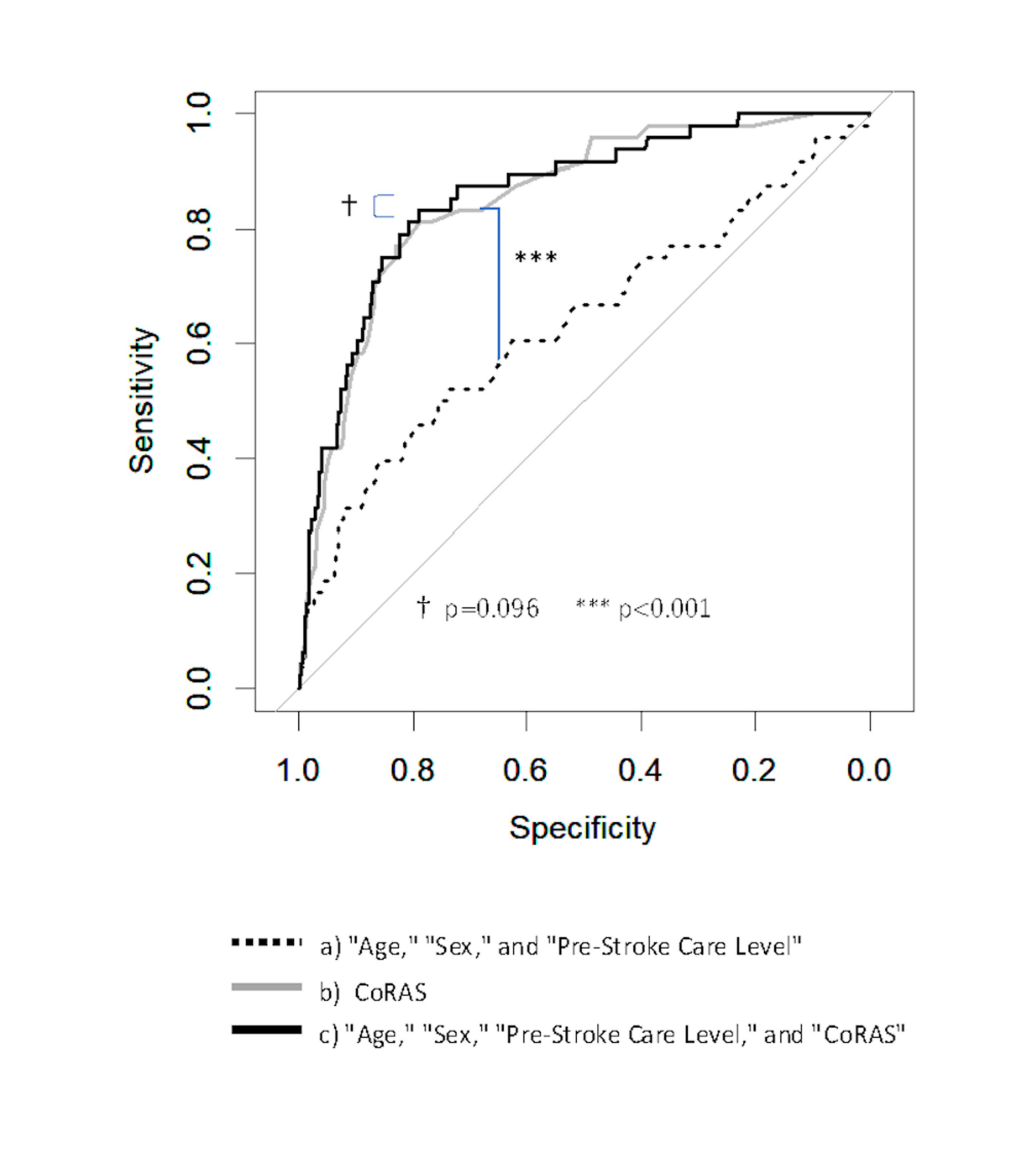
Predictive accuracy of CoRAS for pneumonia CoRAS, Collaborative Respiratory Assessment Score

The AUC and AIC values were as follows: a) 0.636 (95% confidence interval: 0.542-0.730) and 332.6 for the model with age, sex, and pre-stroke care level; b) 0.852 (0.798-0.906) and 264.0 for the model with CoRAS alone; and c) 0.860 (0.806-0.915) and 263.6 for the model with all four factors (age, sex, pre-stroke care level, and CoRAS) (Figure 1, Table 7). Significant differences were observed between models a) and c), and between models a) and b). No significant difference was observed between models b) and c).

**Table 7:** ROC curve analysis and AIC values The AUC of the ROC curve was 0.852 for CoRAS alone and 0.860 after adjustment. AIC, Akaike's Information Criterion; AUC, area under the curve; CoRAS, Collaborative Respiratory Assessment Score; ROC, receiver operating characteristic

Model	AUC	95% Confidence Interval	AIC
Age + sex + pre-stroke care level	0.636	0.542–0.730	332.6
CoRAS	0.852	0.798–0.906	264.0
Age + sex + pre-stroke care level + CoRAS	0.860	0.806–0.915	263.6

## Discussion

Efforts to evaluate respiratory risk using scoring systems have long been conducted, primarily in acute care settings, and have demonstrated effectiveness [1-12]. Both acute care facilities and KRWs aim to accurately assess pneumonia risk and implement interventions to prevent adverse outcomes. To enhance the functionality of KRWs, a unique risk assessment score tailored to the differences in patient backgrounds and team operations is necessary. Based on clinical intuition from various disciplines, we developed CoRAS by selecting and weighing parameters.

Several validation steps were required to utilize CoRAS as an indicator. The eight parameters included in CoRAS were selected based on clinical intuition rather than statistical methods. The potential for overlap among similar variables was anticipated, as evidenced by correlation coefficients reaching up to 0.65 among parameters. In such cases, it is crucial to assess multicollinearity [20]. This issue is fundamental to the score’s validity, and we addressed it by accumulating and analyzing data during its practical application. With a maximum VIF of 2.11 among the eight parameters, the validity of combining these parameters into a linear composite score was demonstrated, justifying CoRAS as a single indicator.

The eight parameters, despite allowable multicollinearity, can be viewed as eight linearly independent vectors from a linear algebra perspective. This suggests that the pathology of pneumonia susceptibility may involve a complex interplay of explanatory variables exceeding eight dimensions. If multidisciplinary perspectives can generate eight linearly independent vectors, it reinforces the value of collaborative patient care. However, non-linear relationships, strong confounding factors, and causal relationships among parameters are expected, necessitating careful consideration of interrelationships.

Another inherent challenge with CoRAS lies in the validity of the weightings assigned to each parameter. To address this, we performed analyses after binarizing the eight parameters and excluding weightings, obtaining similar results. Thus, we reported findings based on the original scores.

The adjusted odds ratio for CoRAS was 1.06 per point, illustrating an exponential increase in pneumonia risk with higher scores. Representing pneumonia risk using a score offers two main advantages. First, it allows professionals from different disciplines to intuitively understand and share the magnitude of risk through numerical values. Second, it provides a reference for categorizing patients into low-, moderate-, and high-risk groups. For instance, low-risk patients can generally be considered unlikely to develop pneumonia (specificity: 0.96), while approximately 40% of high-risk patients develop pneumonia. This stratification enables efficient allocation and concentration of staff resources. The scoring system’s advantages are well-utilized in promoting effective multidisciplinary collaboration.

Adopting evaluation indices already in routine use by each discipline offers at least four benefits. First, the consistency of evaluation methods within each discipline ensures high internal validity. Second, it reduces the likelihood of missing data. Third, evaluations are available by the day after admission, facilitating early team conferences. At this point, comprehensive evaluations and shared understanding of patient conditions enable discussions on intervention strategies. Fourth, CoRAS fosters awareness among staff regarding their discipline’s contribution to overall risk, encouraging role consciousness within the multidisciplinary team. Thus, CoRAS effectively ensures the quality of information while promoting efficient multidisciplinary collaboration, a defining feature of this tool.

When assessing the predictive accuracy of CoRAS using ROC curve analysis [21], the AUC for CoRAS alone was 0.85. Compared to existing scores reported for acute care settings (AUC: 0.6-0.9) [3-6,8-10,12,13,16,18], this performance is comparable. However, KRWs have more comprehensive and stable patient data than acute care settings, leaving room for improved accuracy. Despite adjusting for age, sex, and pre-stroke care level, the AUC remained 0.86, with minimal improvement in AIC. Enhancing predictive accuracy remains a significant challenge.

Improving risk assessment precision requires reevaluating parameter selection. Among the eight CoRAS parameters, SpO₂ did not show significant differences in univariate analyses, suggesting it may not be an effective parameter. However, its inclusion reflects the clinical significance perceived by the staff, and it is one of the linearly independent parameters. In non-stroke populations, it may play a different role. The interrelationships among parameters are complex and unresolved, necessitating further scrutiny before excluding SpO₂ or other parameters. This highlights the broader challenge of refining parameter selection to improve predictive accuracy.

This study has several limitations. While it focused on stroke patients, future research should include other disease groups. Additional tasks include examining interrelationships among parameters, refining parameter selection, and scientifically validating weightings. This study is retrospective and single-center in nature and does not include comparisons with other established pneumonia risk scores. Therefore, further prospective, multicenter studies and external validation are necessary before CoRAS can be broadly implemented.

CoRAS is a respiratory assessment score developed with input from multiple disciplines, reflecting the collaborative spirit essential in KRWs. While this study demonstrated its utility in evaluating pneumonia risk in stroke patients, its potential for high descriptive power and collaboration-promoting effects suggests broader applicability, despite the challenges it faces.

## Conclusions

We devised CoRAS, a respiratory assessment score developed with input from multiple disciplines, and examined its validity. It has been proven to be a valuable tool for predicting pneumonia in stroke patients during their KRW stay. By integrating perspectives and expertise from multiple healthcare disciplines, CoRAS facilitates early identification of pneumonia risk, enabling coordinated preventive interventions. However, this study is a retrospective and single-center study, and it does not benchmark CoRAS against other pneumonia risk scores. Further prospective and multicenter studies, along with external validation, are essential before CoRAS can be widely implemented.
